# Clustered Cases of *Oestrus ovis* Ophthalmomyiasis after 3-Week Festival, Marseille, France, 2013

**DOI:** 10.3201/eid2102.140974

**Published:** 2015-02

**Authors:** Lucas Bonzon, Isabelle Toga, Martine Piarroux, Renaud Piarroux

**Affiliations:** Parasitology Hôpital de la Timone, Marseilles, France (L. Bonzon, I. Toga, R. Piarroux);; Aix-Marseille University, Marseilles (M. Piarroux, R. Piarroux, L. Bonzon)

**Keywords:** *Oestrus ovis*, myiasis, ophthalmology, outbreaks, Mediterranean region, zoonoses, parasites

**To the Editor:** Ophthalmomyiasis is a zoonosis generally caused by *Oestrus ovis*, a fly that lays eggs on the eye of its host. The hatched larvae cause irritation, and left untreated, the infestation can lead to blindness (*1*). The disease is rare and is mainly reported as sporadic cases in pastoral areas where the population is in close contact with common reproductive hosts of the fly, such as sheep and goats in the Middle East (*2*), Southeast Asia (*3*,*4*), and the Mediterranean Basin (*5*,*6*). Only limited ophthalmomyiasis outbreaks have been reported around the Mediterranean Sea (*7*). A century ago, the Provence region of southern France was a pastoral area, where twice a year, large herds of sheep migrated between the pastures in the mountains north of the region and the grassland plains in the southwest. These migrations were termed *transhumance*. In 2013, Marseille metropolis, the largest urban area in Provence, was chosen as the yearly “European Capital of Culture.” In this context, from May 17 to June 9, a large-scale *transhumance* event took place, which featured the gathering of huge flocks of sheep (Figure) that had passed through many towns in the vicinity of Marseille. *La transhumance* culminated in a parade through downtown Marseille, where 600 horseback riders converged with flocks of 3,000 sheep and goats, and >300,000 spectators gathered. 

**Figure:**
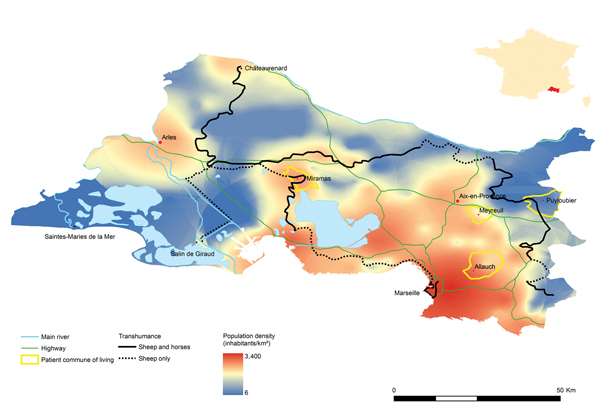
Map of *la Transhumance* routes and locations of *Oestrus ovis* ophthalmomyiasis case-patients in areas surrounding Marseilles, France, 2013. Inset shows location of the area in France.

From the last week of June through the third week of July, 4 cases of ophthalmomyiasis were reported in the area surrounding Marseille (Figure). Only 1 case had occurred in the region during the previous 5 years. The first case-patient was a 45-year-old female teacher, who lived and worked in Allauch, located in the immediate suburbs, 21 km (≈13 mi) east of Marseille. On June 25, while on the school playground, she described feeling a fly hit her right eye. In the evening, itching and irritation of the eye prompted her to seek referral to an ophthalmologic emergency center. Examination concluded the presence of small mobile larvae inside the eye, which were identified as *O. ovis* (Technical Appendix). 

The second case-patient was a retired female farmer, 67 years of age, living near Puyloubier, 49 km (≈30 miles) north of Marseille, who reported being stung on the eye by an insect during the morning of July 6. In the afternoon, the eye became painfully inflamed. On the next day, an ophthalmologist performed an excision and extracted *O. ovis* larvae from the eye. 

The third case-patient was a 43-year-old female nurse’s aide residing in Meyreuil, which is ≈30 km (≈18 mi) north of Marseille. On July 13, while she was on her terrace, she described ocular trauma by a fly. The next day, she sensed a foreign body in her eye, and she consulted an ophthalmologist. A simple excision led to the identification of an *O. ovis* larva. 

The final case-patient was a 28-year-old male mason. On July 22, while working in Miramas, approximately 63 km (≈39 miles) he experienced trauma to his left eye. As the pain persisted, he consulted an ophthalmology facility that same evening. On examination, the presence of an *O. ovis* larva was confirmed, and an ablation was performed. All patients recovered without consequences.

*O. ovis*, also called sheep nasal botfly, is a fly of the class Insecta, order Diptera and family *Oestridae*. It is a cosmopolitan parasite that infects the nasal sinuses of sheep and goats. During the summer and early autumn, the adult female flies are active, laying and retaining eggs until they hatch. The fly then ejects many first-instar larvae onto the nostril of the host. The *O. ovis* larvae grow in the mucus of the nasal sinus until mature; they are then released from the nostrils when the infected host sneezes (*8*). The larvae pupate in the soil for 4–8 weeks, form a chrysalis, in which they morph into adults, and then emerge. Occasionally, *O. ovis* can infest humans, which become an intermediate accidental host (*7*). The 4 cases of ophthalmomyiasis described in this report occurred in a restricted area during a 4-week period, which corresponds exactly to the time and location of *la Transhumance*, taking into account the 4- to 8-week time lag required for the maturation of larvae into adults. Three of these cases were directly referred to our laboratory, the regional referent parasitology laboratory. The fourth case was reported by an ophthalmology emergency unit. Note that ophthalmomyiasis is rare in Marseille; during the 5 years before *la Transhumance* of 2013, only a single case had been diagnosed in the area.

Overall, this report reminds us that bringing a large group of livestock into contact with a dense urban population may enhance the risk for transmission of zoonoses. The transmitted zoonosis in this case was oestrosis, a benign condition that can sometimes progress to blindness if untreated. However, other much more severe air-transmitted zoonotic diseases associated with sheep and goats, such as Q fever, could have been transmitted (*9*). Without questioning the organization of such an event, which the community considers to be important from a cultural and economic point of view, public health authorities should consider and anticipate as much as possible the potential sanitary consequences of such a gathering and prepare medical staff for the potential occurrence of unfamiliar diseases.

Technical Appendix *Oestrus ovis* larva on the eye of case-patient 1 and after collection.
