# Supplementary material for: Clustered Cases of Oestrus ovis Ophthalmomyiasis after 3-Week Festival, Marseille, France, 2013
Source: Emerg Infect Dis. 2015 Feb;21(2):375–7. doi: 10.3201/eid2102.140974 (PMC4313882; doi:10.3201/eid2102.140974)
Supplement: Technical Appendix — Oestrus ovis larva on the eye of case-patient 1 and after collection. [file 14-0974-Techapp-s1.pdf]

Article DOI: http://dx.doi.org/10.3201/eid2102.140974

Clustered Cases of *Oestrus ovis* Ophthalmomyiasis after Three-Week Festival, Marseille, France, 2013

Technical Appendix

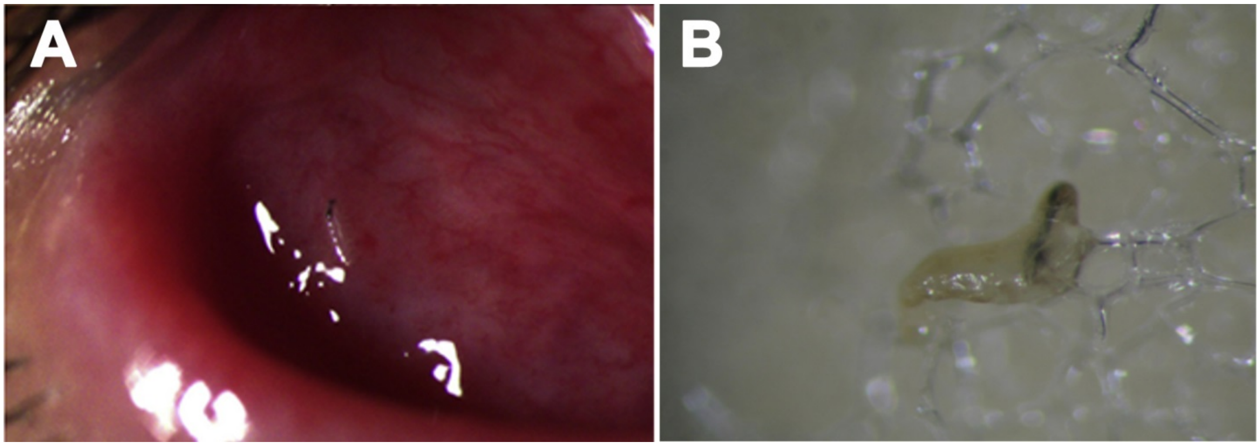

Technical Appendix Figure. *Oestrus ovis* larva on the eye of case-patient 1 and after collection. A) *Fornix conjunctiva* of case-patient 1 showing chemosis (conjunctival edema) and larva of *Oestrus ovis.* B) Larva collected from *Fornix conjunctiva* on swab.
